# Molecular epidemiology and genetic variation analyses of porcine circovirus type 2 isolated from Yunnan Province in China from 2016-2019

**DOI:** 10.1186/s12917-020-02304-8

**Published:** 2020-03-23

**Authors:** Nianci Lv, Li Zhu, Wengui Li, Zhilan Li, Qisheng Qian, Tianyu Zhang, Lu Liu, Jinmei Hong, Xiaolin Zheng, Yuan Wang, Yifang Zhang, Jun Chai

**Affiliations:** College of Animal Medicine, Yunnan Agricultural University, Kunming, 650201 China

**Keywords:** PCV2, Epidemiological survey, Genetic evolution analysis

## Abstract

**Background:**

Porcine circovirus type 2 (PCV2) is the causative agent of porcine circovirus-associated disease (PCVAD). Its prevalence in swine herds was first reported in China in 2000. PCV2 infection causes immunosuppression that leads to multiple diseases, causing serious economic problems for the swine industry in China. Since information on the genetic variation of PCV2 in Yunnan province is limited, this study aims to investigate the molecular epidemiological and evolutionary characteristics of PCV2 from 2016 to 2019.

**Methods:**

A total of 279 clinical samples were collected from different regions of Yunnan between 2016 to 2019, and PCV2 was detected by PCR. We then amplified full genomes from the positive samples, and the sequences were analysed for homology and genetic evolution.

**Results:**

Overall, 60.93% (170/279) of the screened swine herd samples were positive for PCV2. We sequenced 15 Yunnan province PCV2 strains from positive samples. Analyses of the complete genomes and Cap genes led to the classification of the 15 Yunnan PCV2 strains into PCV2a (2 of 15), PCV2b (1of 15) and PCV2d (12 of 15). All strains shared 94.3–99.9% of their identities with the nucleotide sequences of complete genomes in this study and shared 94.2–99.9% identity with the reference sequences. All strains share 89.4–100% and 86.8–100% of their identities with the nucleotide and amino acid (aa) sequences of Cap, respectively.

**Conclusions:**

The results of this study provide evidence that PCV2a, PCV2b and PCV2d genotypes coexisted in Yunnan Province from 2016 to 2019, and the priority prevalence genotype was PCV2d. The data provide evidence for the increased genetic diversity and insights into the molecular epidemiology of PCV2. This study also provides basic data for the Yunnan province PCV2 molecular epidemiological survey and accumulates effective materials for the development of PCV2 vaccines.

## Background

Porcine circovirus type 2 (PCV2), a member of the family *Circoviridae*, is a small, non-enveloped virus with a circular, single-stranded DNA genome. It has an icosahedral symmetrical structure with a diameter of approximately 17 nm [1]. PCV2 is the causative agent of porcine circovirus-associated disease (PCVAD), which manifests as postweaning multisystemic wasting syndrome (PMWS), reproductive failure, porcine dermatitis and nephropathy syndrome (PDNS), congenital tremors (CT), porcine respiratory disease complex (PRDC), enteritis, and proliferative and necrotizing pneumonia (PNP), among other conditions [2]. Since its identification in the 1970s, PCV2 has become prevalent globally. PCV2 infection normally causes immunosuppression and reduced immunity by damaging the immune system, which leads to co-infections with other pathogens, such as Porcine Reproductive and Respiratory Syndrome Virus (PRRSV), CSFV and Porcine Pseudorabies Virus (PRV), usually resulting in worse conditions, making the requirements for prevention and control more serious [3]. PCV2 infection has caused huge economic losses in pig production in China and worldwide and is recognized as one of the most important pig diseases.

The genome of PCV2 contains 1766–1768 nucleotides, with eleven overlapped and nested open reading frames (ORFs). Six ORFs (ORF1–6) encode proteins that have been identified from these overlapped and nested ORFs [4, 5]. The viral genome contains two major open reading frames: ORF1 and ORF2. ORF1 encodes two proteins associated with replication, designated as Rep and Rep’ [6]. ORF2 encodes the capsid (Cap) protein. The Cap protein is the only structural protein of PCV2 virus that contains immunologically important epitopes associated with virus neutralization. Therefore, the ORF2 and Cap proteins are commonly used for PCV2 phylogeny, epidemiology, and genetic diversity analyses [3]. PCV2 may evade the immune response in the form of mutations because of the immune pressure due to vaccination and may affect the antigenicity and immunogenicity of the Cap protein. Huang found that after a single amino acid change in the PCV2 Cap protein, a strain cannot be neutralized by an antibody known to have neutralizing capacity [7]. PCV2 maintains evolutionary dynamics closer to single-stranded RNA viruses than double-stranded DNA viruses because the rate of nucleotide substitution for PCV2 has been estimated to be 1.2 × 10^− 3^ sites per year, which is the highest recorded substitution rate for a single-stranded DNA virus [8].

Since the first isolation of the PCV2 virus in 1998, PCV2 strains have been classified into five genotypes (PCV2a, PCV2b, PCV2c, PCV2d and PCV2e) based on phylogenetic analyses of genomes and ORF2 sequences [9]. PCV2a is the first genotype to be identified, and PCV2a and PCV2b are distributed worldwide. PCV2a can now be further subdivided into five (2A-2E) and three (1A-1C) clusters [10, 11]. Since 2003, there has been an observable worldwide genotypic shift, with PCV2b replacing PCV2a and becoming the predominant genotype. The PCV2d genotype has recently become a popular genotype in the world and can be subdivided into two clusters (2d-1 and 2d-2) [11, 12]. PCV2c was detected only in Denmark in archived swine serum samples [13]. PCV2e is a new genotype that has been circulating in the USA since 2015, the ORF2 sequences of which have 12 or 15 additional nts (CTT/TCTTATATGTAA) compared to those of PCV2a-PCV2d. PCV2e was reported in China for the first time in 2017 [14, 15]. Intermediate and new genotypes have been proposed, with increasing numbers of sequences available in recent years. For example, the recombinant cluster that recombines between PCV2a and PCV2b strains can occur in the Cap protein-coding region through different patterns and yield different recombinants [16]. In 2018, results from a retrospective analysis showed that a new genotype, PCV2f, was tracked to samples collected in 1996 in China [17]. Up to now, there is no specific treatment available for PCV2 infection and PCVADs. Vaccination has proved to be the best choice to prevent PCV2 infection in the field. However, with its high evolution rate, the virus’ predominant genotype is constantly changing. This leads to the appearance of new genotypes and increased difficulty for vaccine development, making the prevention and treatment of PCV2 more difficult. Thus, investigation of the antigens and capsid structure variations of the virus is critical to improve diagnosis, new vaccine design, and our understanding of the PCV2 pathogenesis of this disease.

In the past few years, other studies have provided remarkable knowledge of PCV2 epidemiology and genetic variation. Nevertheless, there is limited information on the genetic variation of PCV2 in Yunnan province. In this study, we investigated the prevalence of PCV2 based on 279 clinical pig samples from 11 different regions of Yunnan from 2016 to 2019. The objectives of the study were to investigate the epidemiological and evolutionary characteristics of PCV2 in Yunnan province from 2016 to 2019. The results of this study may enrich the epidemiological data of PCV2 in our province and provide reference information for the comprehensive prevention and control of PCV2.

## Results

### PCV2 detection

A total of 279 pig tissue samples from pig farms in different regions of Yunnan were screened for PCV2 by PCR from 2016 to 2019. The length of the amplified products was 549 bp of the PCV2 genome. A total of 170 samples were PCV2-positive (60.93%) (Table 1). The infection rate varied between different ages, with the highest found in aborted pigs, while milking sows had the lowest infection rate, and fattening pigs were similar to nursery pigs (Table 2).

**Table 1:** PCV2 infections in Yunnan from 2016 to 2019

Geographic origin	Positive samples/total samples				Total	Positive rate (%)
	2016	2017	2018	2019		
Kunming	6/10	17/27	13/16	8/17	44/68	64.71
Qujing	3/8	19/30	19/29	10/15	51/82	63.20
Yuxi	0/1	5/12	5/7	1/1	11/21	57.14
Lijiang	–	2/3	4/7	–	6/10	60.00
Dali	–	4/7	1/1	–	5/8	62.50
Xishuangbanna	–	7/13	–	2/2	9/15	60.00
DeHong	4/6	13/27	5/8	–	22/41	53.65
HongHe	2/2	3/5	11/20	–	16/27	59.26
Chuxiong	–	1/2	–	1/1	2/3	66.67
Wenshan	–	–	3/5	1/2	4/7	57.14
Total	15/24	71/126	61/93	23/36	170/279	/
Positive rate (%)	62.50	56.35	65.59	63.89	60.93	/

-: Indicates no sample collected

**Table 2:** PCV2 infections in different ages of pigs from Yunnan from 2016 to 2019

Ages of pigs	Positive samples	Total samples	Positive rate (%)
Nursery pigs	51	82	62.20
Fattening pigs	84	134	62.69
Milking sows	10	26	38.46
Aborted pigs	25	37	67.57
Total	170	279	60.93

### Genomic sequences

Fifteen full genomes of PCV2 isolates from positive samples collected from 7 prefectures of Yunnan province were sequenced. These genome sequences were submitted to the GenBank database under the following accession numbers: MH151168, MH645914, MH656967, MH593263, MH620789, MK405696-MK405701, MK424113-MK424115, and MK987069.

### Phylogenetic analysis of PCV2 using complete genome and ORF2 sequences

**Fig. 1:**
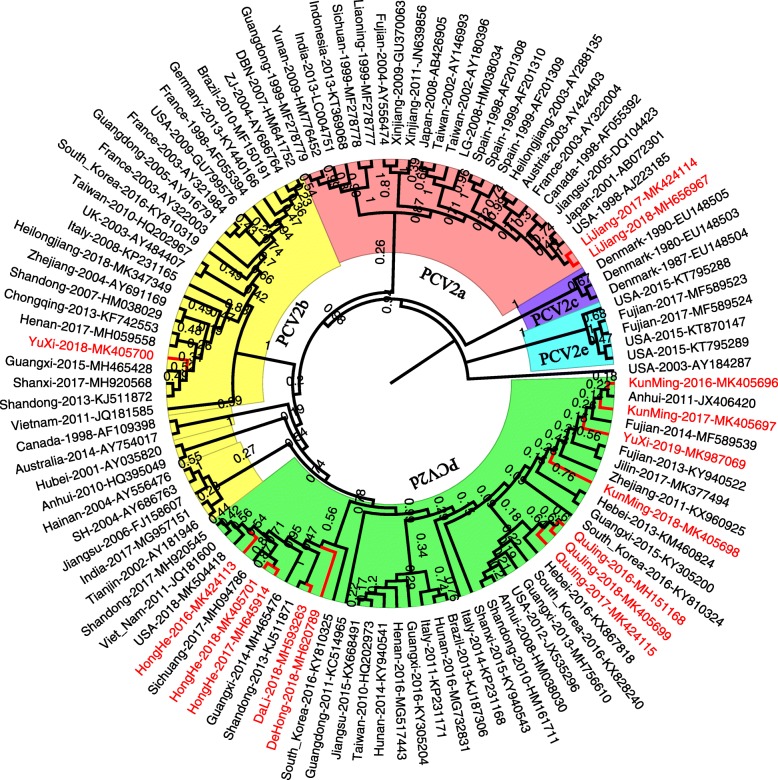
Phylogenetic analysis based on complete genome sequences obtained in this study together with the reference sequences from GenBank. The neighbor-joining tree was constructed by molecular evolutionary genetics analysis (MEGA) software version 7.0 with the evolutionary distances were computed using the p-distance method. A PCV1 sequence provided an outgroup to root the tree. The PCV2 strains obtained in the present study are indicated with red

A phylogenetic tree based on genomes of the 15 PCV2 strains was constructed together with 95 reference sequences representing genotypes from China and other countries. The phylogenetic trees displayed similar topologies in both neighbour-joining (NJ) and maximum-likelihood (ML) methods, and five main clades were delimited with porcine circovirus type 1 (PCV1) as an outgroup. Phylogenetic analysis showed that Yunnan PCV2 strains were classified into three subtypes: PCV2a, PCV2b and PCV2d (Fig. 1). Two strains belonged to the PCV2a genotype, one strain belonged to the PCV2b genotype and other strains belonged to the PCV2d genotype; the genotype proportions of isolated strains were 13.3, 6.7 and 80% respectively. We did not find the 2c and 2e genotypes in Yunnan, and the NJ tree based on ORF2 gene sequences displayed similar results with that of the complete PCV2 genome.

### Sequence alignment

The genome sizes of 13 PCV2 strains were 1767 nucleotides, and 2 isolates were 1768 nucleotides in length. The complete genomes and ORF2 genes were analysed to detect the relationships between and within the different genotypes of the 15 Yunnan PCV2 isolates (Table 3). We found that all strains shared 94.3–99.9% nucleotide sequence identity for the whole genome within the different genotypes of 15 Yunnan PCV2 strains and 94.3–99.9% identity with reference sequences. Among the PCV2a, PCV2b, and PCV2d strains sequenced in this study, genotypes PCV2b and PCV2d had the highest nucleotide identity (96.0–97.6%), and PCV2a was more different from PCV2d in terms of identity (94.3–95.0%). When compared with two vaccine strains, SH and LG (AY686763 and HM038034), all nucleotide homology levels of the Yunnan strains were 94.7–98% and 95.1–96.7%, respectively.

**Table 3:** Nucleotide identity of 15 Yunnan PCV2 complete genomes and ORF2 gene sequences within and between genotypes

Nucleotide identity within genotypes, complete genome (%)	Genotype	Nucleotide identity (%) between PCV2 genotypes			Nucleotide identity within genotypes, ORF2 gene (%)
		PCV2a	PCV2b	PCV2d	
99.7	PCV2a	–	91.7	89.7–90.5	99.7
–	PCV2b	95.2–95.5	–	94.3–95.1	–
98.2–99.9	PCV2d	94.3–95.1	96.0–97.6	–	99–100

The upper right indicates the nucleotide sequence identity of ORF2

The lower left indicates the nucleotide sequence identity of complete genome

The results of ORF2 sequence analysis showed that the homology of the ORF2 gene of Yunnan strains ranged from 89.7–100%, and the homology with the ORF2 genes of domestic and foreign reference strains ranged from 89.4–100%, similar to the homology analysis results based on complete genome sequence analysis. In contrast, the homology of strains between different genotypes was quite different; for instance, the homology between PCV2a and PCV2d ranged from 89.5–92.6%.

### Amino acid analysis of ORF2

**Fig. 2:**
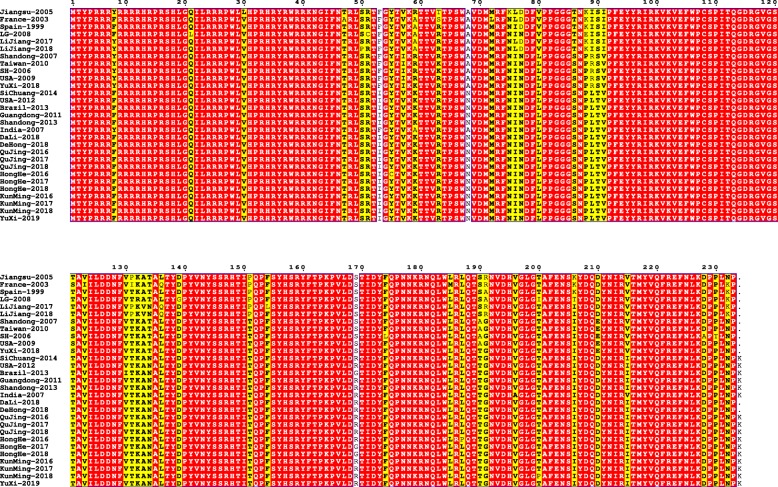
Alignment of Cap protein sequences of 15 Yunnan isolates and 14 reference sequences. The Sequences Jiangsu-2005 to LiJiang-2018 correspond to PCV2a, Shandong-2007 to YuXi-2018 correspond to PCV2b, and SiChuang-2014 to YuXi-2019 correspond to PCV2d. The first row represents the site of the amino acid

The lengths of ORF2 of the Yunnan PCV2 isolate were 702 nts (PCV2a, PCV2b or PCV2d) and 705 nts (PCV2d), encoding Cap proteins of 233 and 234 amino acids, respectively. Further analysis of the Cap protein sequences of the 15 Yunnan PCV2 strains (Fig. 2) indicated that 2 of the 15 isolates (LiJiang-2017 and LiJiang-2018) corresponded to PCV2a, with three unique amino acid mutations at residues 133 (A133V), 134 (T134N), and 206 (K206I), and unique amino acid mutations at residue 154 (F154L) in LiJiang-2017 and residue 50 (S50P) in LiJiang-2018. The Yuxi-2018 strain corresponded to PCV2b. In addition, Yuxi-2018 harboured a unique amino acid mutation at residue 59 (R59K). Twelve isolates corresponded to PCV2d. As the result of a mutation at the stop codon, 9 of the 15 strains had 234 amino acids, and two strains, DaLi-2018 and DeHong-2018, encoded only 233 amino acids. Several aa changes occurred in some of the strains of PCV2d in this study, such as the 53 (Y53C),187 (L187P) amino acid mutations in HongHe-2016. 169 (R169G) mutations in HongHe-2017 and HongHe-2018, and 200 (T200P) mutations in KunMing-2018.

## Discussion

### PCV2 infection in Yunnan province

Porcine circovirus type 2 is the causative agent of PCVAD and is one of the most notorious diseases in the global swine industry. Vaccination pressure, natural selection, and the international pig trade are all responsible for the high genetic diversity of PCV2 in China [18]. PCV2 is widespread in herds worldwide. Though PCV2 infection alone does not cause clinical disease, the resulting autoimmune inhibition still leads to co-infection; thus, the virus is responsible for multiple diseases. PCV2 infection has caused serious economic problems for the swine industry in China [19]. In this study, 279 samples collected from 2016 to 2019 were screened for PCV2 by PCR, with a positive rate of 60.93%. Moreover, the infection rate of PCV2 increased from 2016 to 2019. Despite the widespread use of PCV2 vaccines, clinical and subclinical infections of PCV2 were still present in Yunnan swine herds.

### Genomic sequence analysis

PCV2 is highly prevalent in pig herds, all currently recognized genotypes have been found in China except for PCV2c [20]. PCV2a has been prevalent in pig herds since PCV2 infection was first reported in China in 2000, and the predominant genotype has shifted from PCV2a to PCV2b. In more recent times, PCV2d has become the new emerging major genotype circulating in China [16]. PCV2d can be further divided into two main groups: PCV2d-1 and PCV2d-2. In this study, we found that PCV2d-2 has replaced PCV2b and become the predominant PCV2 genotype [21]. Phylogenetic analysis showed that the co-infection of multiple PCV2 genotypes (2a, 2b, 2d) is common in Yunnan pig herds, and the PCV2d strain is the main epidemic genotype, accounting for 80% (12/15) of the isolates in Yunnan. The genetic diversity of PCV2 is continuously increasing, and the cycle of the three genotypes continues in the herd. These results indicate that the prevalence of PCV2 in Yunnan has been complicated in recent years.

### Sequence alignment of ORF2

The results of sequence alignment revealed that the nucleotide sequences of Yunnan PCV2 isolates showed significant similarities at different times in the same region, with some similarities reaching 99.9%. Strains isolated from sites in close proximity to each other also have higher homology. ORF2 encodes the capsid protein, which is the only structural protein of the PCV2 virus that contains immunologically important epitopes associated with virus neutralization [22]. Lower nucleotide sequence identities and amino acid homology with vaccine strains will result in noneffective cross-protection and immune failure [23]. PCV2b isolates presented a high amino acid sequence identity of 97.4% with vaccine strain SH (HM038027), and PCV2a isolates presented an amino acid sequence identity of 93.1% with vaccine strain LG (M038034). In contrast, PCV2b isolates presented an amino acid sequence identity ranging from 89.7–92.7% with two vaccine strains. Interestingly, the genotype shift from PCV2b to PCV2d appeared mainly in pig herds vaccinated with a PCV2 vaccine, and the homology is similar between PCV2d and PCV2b, suggesting that PCV2d originated from PCV2b.

### Amino acid analysis of ORF2

ORF2-encoded capsid proteins of different PCV2 genotypes can be distinguished according to the typical motifs in Cap protein. The typical motif TNKISI is present in 86–91 sites in PCV2a. The motifs 190–191/206/210 S/PNPRSV and A/TGIE are present in PCV2b. SNPLTV and TGID motifs are present in most PCV2d strains [24]. Additionally, the amino acid epitopes 50–81, 113–134, 161–208, and 227–233 in the Cap protein of PCV2 have been previously identified [25]. Alanine at position 59, threonine at position 190, and glutamic acid at position 191 are parts of a conformational neutralizing epitope [7, 26]. Asparagine at position 77 and isoleucine at position 206 are key residues and essential for antibody recognition [27]. According to a previous report, if an amino acid in an epitope mutates, the mutated virus cannot be recognized by the epitope’s antibody [28]. Meanwhile, compared with the traditional PCV2a/PCV2b strain, the PCV2d strain contains seven unique amino acids (53I, 59 K, 68 N, 89 L, 90 T, 134 N, 169R) in the Cap protein sequence, and these amino acids are mostly distributed along the surface of the viral nucleocapsid. This new surface conformation also renders the antibodies raised against the SH and LG vaccines unable to neutralize the virus [29]. In the present study, several aa mutations occurred at epitopes in the Cap protein. For the isolates Lijiang-2017 and Lijiang-2018, mutations occurred at positions 133 (A133V), 134 (T134N) and 206 (I206K). Although Lijiang-2017 and Lijiang-2018 belong to PCV2a, these aa mutations are consistent with PCV2d unique amino acid sequences and occurred at epitope-associated positions. Yuxi-2018 isolates belong to PCV2b; a mutation at position 59 (R59K) is consistent with PCV2d. These aa mutations may be responsible for contributing to genotypic variation in PCV2. In addition, several PCV2d isolates also substituted mutations at positions 53 (Y53C) and 187 (L187P) in HongHe-2016, 169 (R169G) in HongHe-2017 and HongHe-2018, and 200 (T200P) in KunMing-2018. These aa mutations at epitope-associated positions may be responsible for PCV2 escaping from the immune defence of the host and promoting the spread of PCV2 strains among pig populations. Although the widespread use of vaccines can control PCVAD, PCV2 is still present in pig herds, which suggests that some changes might have occurred on the viruses. PCV2 escapes the host immune system by continuously changing its antigenic properties. Mutations at the positions between different genotypes of the isolates are positively selected under pressure, suggesting that amino acid changes at these sites are beneficial for the virus and may contribute to the escape of PCV2 from the host immune system.

## Conclusions

The results of this study provide evidence that PCV2a, PCV2b, and PCV2d genotypes coexisted in Yunnan province from 2016 to 2019, and the most prevalent genotype was PCV2d. The data provide evidence for increased genetic diversity and insights into the molecular epidemiology of PCV2. These data indicate that the prevalence of PCV2 in Yunnan province in recent years is complicated. Mutations occurred at epitope-associated positions, suggesting that the aa changes may have contributed to the escape of PCV2. This study also provides PCV2 prevalence and genetic variation data for Yunnan province and will benefit the development of new effective PCV2 vaccines.

## Methods

### Samples

During disease outbreaks, both blood and tissue samples were collected through necropsy of dead bodies in live clinical cases, only blood samples were collected. A total of 279 clinical samples (blood, lungs, kidneys, livers, and lymph nodes) were collected from 17 different-sized pig farms with 100–1000 sows in 7 regions in Yunnan province, China, from 2016 to 2019. All clinical samples were from pigs of different ages, including Nursery pigs, Fattening pigs, Milking sows, Breeding boars and Aborted pigs.

The experimental protocols were approved by the Animal Ethics Committee at Yunnan Agricultural University (permission code: YAUACUC2016; date of publication: July 10, 2016), and animals were handled according to the animal ethics guidelines.

### Detection of PCV2

Total viral DNA was extracted directly from sera and tissue samples using the DNeasy Tissue Kit (TaKaRa) according to the manufacturer’s instructions. Two pairs of primers were used to detect PCV2 (F1 ACCGTTACCGCTGGAGAAGGAAAAA, R1 TGGTTACACGGATATTGTAGTCCTG) and to amplify the whole 1.7-kb genome (F2 ATCCACGGAGGAAGGGGGCCAGTT, R2 GTGGATTGTTCTGTAGCATTCTTCCA). The PCRs in a total volume of 25 μl contained 12.5 μl of 2 × Easy Taq PCR Mix (TaKaRa),1 μl of extracted DNA, 1 μl of F1, 1 μl of F2, and 9.5 μl of ddH_2_O. PCR amplification was initiated at a predenaturation stage of 94 °C for 5 min, followed by 34 cycles of denaturation at 94 °C for 30 s, annealing at 62 °C for 30 s, and extension at 72 °C for 40 s. PCR products were analysed via 1% agarose gel electrophoresis and photographed using a gel imaging system. The amplified products were 549 bp in length in positive samples.

### qPCR and genome sequencing protocols

Strong-positive samples from PCV2-positive samples were selected by qPCR, and the standard curve was established. The qPCR amplification of PCV2 was carried out in 20-μL reaction mixtures containing 10 μL of TB Green II (TaKaRa), 0.8 μL of Forward primer (F), 0.8 μL of Reverse primer (R), 0.4 μL of ROX and 50 ng of DNA template. A strong linear relationship was also evident, with an R2 of 0.999, and the E was 100.91%. The linear relationship between Ct and the copy number was calculated as: Ct = − 3.3X+ 31.35. Based on geographic region, prevalence time and positive rate, representative strains were selected from positive samples for genome sequencing. The selected positive samples used primers F2/R2 to amplify the genome. Subsequently, positive PCR products 1767 nts in length were purified using the DNA Glue recovery kit (TaKaRa). The purified PCV2 genomic DNA products were ligated into the PMD19-T vector (TaKaRa) and then used to transform *Escherichia coli* DH5α competent cells (TaKaRa). Positive bacterial suspensions were sent to Shanghai Huada Corporation for sequencing. Whole genome was sequenced twice, and the sequence was submitted to the NCBI GenBank database.

### Phylogenetic analysis

Fifteen complete genomes of PCV2 isolates were identified in Yunnan province from 2016 to 2019. Ninety-five representative PCV2 sequences, including PCV2a, PCV2b, PCV2c, PCV2d, and PCV2e, downloaded from GenBank, were used for phylogenetic analyses. Phylogenetic trees based on complete genomes were constructed using the neighbour joining (NJ) method with MEGA 7.0, the maximum composite likelihood model, and a bootstrap confidence value of 1000 replicates. Translations of 15 ORF2 nucleotide sequences to amino acid sequences were made using DNAStar software. Multiplex sequencing alignments were performed using ClustalW in the Megalign programme (DNAStar software).

### Amino acid analysis of ORF2

The amino acid sequences of ORF2 were aligned using a multiple sequence aligner in ClustalW of the MEGA 7.0 to identify amino acid alterations.

## Data Availability

All relevant data are within this paper. Accession number of sequences obtained in this study are MH151168, MH645914, MH656967, MH593263, MH620789, MK405696-MK405701, MK4241 13-MK424115 and MK987069.

## Abbreviations

aa: Amino acid
CSFV: Swine fever virus
CT: Congenital tremors
DNA: Deoxyribonucleic Acid
ORFs: Open reading frames
PCR: Polymerase Chain Reaction
PCV2: Porcine circovirus type 2
PCVAD: Porcine circovirus-associated disease
PDNS: Porcine dermatitis and nephropathy syndrome
PMWS: Postweaning multisystemic wasting syndrome
PNP: Proliferative and necrotizing pneumonia
PRDC: Porcine respiratory disease complex
PRRSV: Porcine Reproductive and Respiratory Syndrome Virus
PRV: Porcine Pseudorabies Virus

## References

[1] Hamel AL, Lin LL, Nayar GP (1998). Nucleotide sequence of porcine circovirus associated with postweaning multisystemic wasting syndrome in pigs. J Virol.

[2] Segales J (2012). Porcine circovirus type 2 (PCV2) infections: clinical signs, pathology and laboratory diagnosis. Virus Res.

[3] Karuppannan AK, Opriessnig T (2017). Porcine circovirus type 2 (PCV2) vaccines in the context of current molecular epidemiology. Viruses.

[4] He J, Cao J, Zhou N, Jin Y, Wu J, Zhou J (2013). Identification and functional analysis of the novel ORF4 protein encoded by porcine circovirus type 2. J Virol.

[5] Li D, Wang J, Xu S, Cai S, Ao C, Fang L (2018). Identification and functional analysis of the novel ORF6 protein of porcine circovirus type 2 *in vitro*. Vet Res Commun.

[6] Li W, Liu S, Wang Y, Deng F, Yan W, Yang K (2013). Transcription analysis of the porcine alveolar macrophage response to porcine circovirus type 2. BMC Genomics.

[7] Huang LP, Lu YH, Wei YW, Guo LJ, Liu CM (2011). Identification of one critical amino acid that determines a conformational neutralizing epitope in the capsid protein of porcine circovirus type 2. BMC Microbiol.

[8] Firth C, Charleston MA, Duffy S, Shapiro B, Holmes EC (2009). Insights into the evolutionary history of an emerging livestock pathogen: porcine circovirus 2. J Virol.

[9] Bedolla Lopez F, Trujillo Ortega ME, Mendoza Elvira S, Quintero Ramirez V, Alonso Morales R, Ramirez-Mendoza H (2018). Identification and genotyping of porcine circovirus type II (PCV2) in Mexico. Virus Dis.

[10] Olvera A, Cortey M, Segales J (2007). Molecular evolution of porcine circovirus type 2 genomes: phylogeny and clonality. Virology.

[11] Xiao CT, Halbur PG, Opriessnig T (2015). Global molecular genetic analysis of porcine circovirus type 2 (PCV2) sequences confirms the presence of four main PCV2 genotypes and reveals a rapid increase of PCV2d. J Gen Virol.

[12] Jiang CG, Wang G, Tu YB, Liu YG, Wang SJ, Cai XH (2017). Genetic analysis of porcine circovirus type 2 in China. Arch Virol.

[13] Wang C, Pang VF, Lee F, Huang TS, Lee SH, Lin YJ (2013). Prevalence and genetic variation of porcine circovirus type 2 in Taiwan from 2001 to 2011. Res Vet Sci.

[14] Harmon KM, Gauger PC, Zhang J, Pineyro PE, Dunn DD, Chriswell AJ (2015). Whole-genome sequences of novel porcine circovirus type 2 viruses detected in swine from Mexico and the United States. Genome Announc.

[15] Liu J, Wei C, Dai A, Lin Z, Fan K, Fan J (2018). Detection of PCV2e strains in Southeast China. PeerJ.

[16] Cai L, Ni J, Xia Y, Zi Z, Ning K, Qiu P (2012). Identification of an emerging recombinant cluster in porcine circovirus type 2. Virus Res.

[17] Bao F, Mi S, Luo Q, Guo H, Tu C, Zhu G (2018). Retrospective study of porcine circovirus type 2 infection reveals a novel genotype PCV2f. Transbound Emerg Dis.

[18] Xu PL, Zhao Y, Zheng HH, Tian RB, Han HY, Chen HY (2019). Analysis of genetic variation of porcine circovirus type 2 within pig populations in Central China. Arch Virol.

[19] Ouyang T, Zhang X, Liu X, Ren L (2019). Co-infection of swine with porcine circovirus type 2 and other swine. Viruses.

[20] Wang H, Gu J, Xing G, Qiu X, An S, Wang Y (2019). Genetic diversity of porcine circovirus type 2 in China between 1999-2017. Transbound Emerg Dis.

[21] Xiao CT, Halbur PG, Opriessnig T (2012). Complete genome sequence of a novel porcine circovirus type 2b variant present in cases of vaccine failures in the United States. J Virol.

[22] Ramos N, Porley D, Mirazo S, Castro G, Cabrera K, Lozano A (2017). Molecular study of porcine circovirus type 2 in wild boars and domestic pigs in Uruguay from 2010 to 2014: predominance of recombinant circulating strains. Gene.

[23] Park KH, Oh T, Yang S, Cho H, Kang I, Chae C (2019). Evaluation of a porcine circovirus type 2a (PCV2a) vaccine efficacy against experimental PCV2a, PCV2b, and PCV2d challenge. Vet Microbiol.

[24] Cheung AK (2009). Homologous recombination within the capsid gene of porcine circovirus type 2 subgroup viruses via natural co-infection. Arch Virol.

[25] Trible BR, Kerrigan M, Crossland N, Potter M, Faaberg K, Hesse R (2011). Antibody recognition of porcine circovirus type 2 capsid protein epitopes after vaccination, infection, and disease. Clin Vaccine Immunol.

[26] Saha D, Lefebvre DJ, Ooms K, Huang L, Delputte PL, Van Doorsselaere J (2012). Single amino acid mutations in the capsid switch the neutralization phenotype of porcine circovirus 2. J Gen Virol.

[27] Khayat R, Brunn N, Speir JA, Hardham JM, Ankenbauer RG, Schneemann A (2011). The 2.3-angstrom structure of porcine circovirus 2. J Virol.

[28] Li D, Du Q, Wu B, Li J, Chang L, Zhao X (2017). Immunogenicity of adenovirus vaccines expressing the PCV2 capsid protein in pigs. Vaccine.

[29] Zhan Y, Wang N, Zhu Z, Wang Z, Wang A, Deng Z (2016). In silico analyses of antigenicity and surface structure variation of an emerging porcine circovirus genotype 2b mutant, prevalent in southern China from 2013 to 2015. J Gen Virol.

